# The importance of albumin infusion rate for plasma volume expansion following major abdominal surgery – AIR: study protocol for a randomised controlled trial

**DOI:** 10.1186/s13063-016-1714-5

**Published:** 2016-12-07

**Authors:** Svajunas Statkevicius, Johan Bonnevier, Björn P. Bark, Erik Larsson, Carl M. Öberg, Päivi Kannisto, Bobby Tingstedt, Peter Bentzer

**Affiliations:** 1 Department of Anaesthesia and Intensive Care, Skåne University Hospital, Lund, Sweden; 2 Department of Radiation Physics, Skåne University Hospital, Lund, Sweden; 3 Department of Nephrology, Skåne University Hospital, Lund, Sweden; 4 Department of Gynaecology and Obstetrics, Skåne University Hospital, Lund, Sweden; 5 Department of Surgery, Skåne University Hospital, Lund, Sweden; 6 Department of Anaesthesia and Intensive Care, Helsingborg Hospital and Lund University, 251 87 Helsingborg, Sweden

**Keywords:** Albumin, Infusion rate, Plasma volume expansion, Permeability, Transcapillary escape rate

## Abstract

**Background:**

Administration of fluids to restore normovolaemia is one of the most common therapeutic interventions performed peri-operatively and in the critically ill, but no study has evaluated the importance of infusion rate for the plasma volume-expanding effect of a resuscitation fluid. The present study is designed to test the hypothesis that a slow infusion of resuscitation fluid results in better plasma volume expansion than a rapid infusion.

**Methods/design:**

The study is a single-centre, assessor-blinded, parallel-group, randomised prospective study. Patients over 40 years of age admitted to the post-operative care unit after a Whipple procedure or major gynaecological surgery and presenting with signs of hypovolaemia are eligible for inclusion. Patients are randomised in a 1:1 fashion with no stratification to either rapid (30 minutes) or slow (180 minutes) infusion of 5% albumin at a dose of 10 ml/kg ideal body weight. Plasma volume is measured using ^125^I human serum albumin at baseline (prior to albumin infusion) as well as at 30 minutes and 180 minutes after infusion start. The primary endpoint is change in plasma volume from baseline to 180 minutes after the start of 5% albumin infusion. Secondary endpoints include the integral of plasma volume over time from baseline to 180 minutes after the start of the infusion and transcapillary escape rate of albumin (%/h) from 180 minutes to 240 minutes after the start of albumin infusion. In addition, diuresis, change in central venous oxygen saturation, lactate and blood pressure will be evaluated. A total of 70 patients will be included in the study, and the study has 80% power to detect a difference of 4 ml/kg in plasma volume expansion between the two groups.

**Discussion:**

The present study is the first clinical investigation of the importance of infusion rate for the plasma volume-expanding effect of a resuscitation fluid.

**Trial registration:**

EudraCT identifier: 2013-004446-42. Registration date: 20 December 2013.

ClinicalTrials.gov identifier: NCT02728921. Registration date: 31 March 2016.

**Electronic supplementary material:**

The online version of this article (doi:10.1186/s13063-016-1714-5) contains supplementary material, which is available to authorized users.

## Background

Major surgery initiates a systemic inflammatory response syndrome (SIRS), which disrupts the normal regulation of transcapillary fluid exchange with tissue oedema and hypovolaemia as a consequence. Hypovolaemia will amplify the inflammatory reaction by reducing cardiac output and oxygen delivery, which creates a vicious circle. Fluid therapy is therefore a cornerstone in the peri-operative treatment of patients undergoing major surgery as well as in patients with increased vascular leakage of other aetiologies. However, even if fluid therapy is life-saving, it is also associated with side effects such as further oedema formation, coagulopathy and further endothelial dysfunction. Several studies indicate that these side effects may adversely affect outcome following surgery [1, 2]. Also, in other patient groups with SIRS in the intensive care unit (ICU), it has been shown that fluid overload is associated with increased incidence of respiratory failure and worse outcome [3–6].

From a clinical perspective, it is therefore important that the fluid administered to antagonize hypovolaemia remains intravascular as far as possible. Colloids are macromolecules for which the vessel wall has a low permeability, and proponents of colloids argue that less volume is required for equal plasma volume compared with crystalloids. However, extravasation of colloids not only is a function of the vessel wall permeability but also is dependent on the volume of fluid that is filtered across the capillary wall, which in turn depends on the transcapillary hydrostatic pressure [7]. In addition, transient hypervolaemia induced by rapid administration of colloids may induce increased release of the permeability-increasing diuretic agent atrial natriuretic peptide and components of the endothelial glycocalyx [8, 9].

Taken together, these data indicate that slow administration may reduce extravasation of colloids by minimizing transient hypervolaemia and transient increases in hydrostatic pressure. This hypothesis is supported by results derived from two animal model studies of sepsis-induced systemic inflammatory response showing that the plasma volume expansion was greater in animals randomised to receive a slow infusion than in those randomised to receive a rapid infusion of the same volume of colloid [10, 11].

Fluid administration is one of the most common therapeutic interventions in ICUs and in post-operative units around the world [12], and a fluid bolus is a recommended method to assess fluid responsiveness. However, to date, no study has addressed the importance of infusion rates in a clinical setting. Although rapid correction of hypovolaemia makes intuitive sense, the need for further knowledge in this aspect of fluid resuscitation was highlighted by the recent Fluid Expansion as Supportive Therapy trial, which showed a surprising increase in mortality following a rapid administration of resuscitation fluids compared with less aggressive fluid resuscitation [13]. Based on these considerations, the primary objective of the present study is to test the hypothesis that plasma volume expansion of a given volume of colloid is greater if fluid is administered slowly rather than rapidly.

## Methods/design

**Fig. 1:**
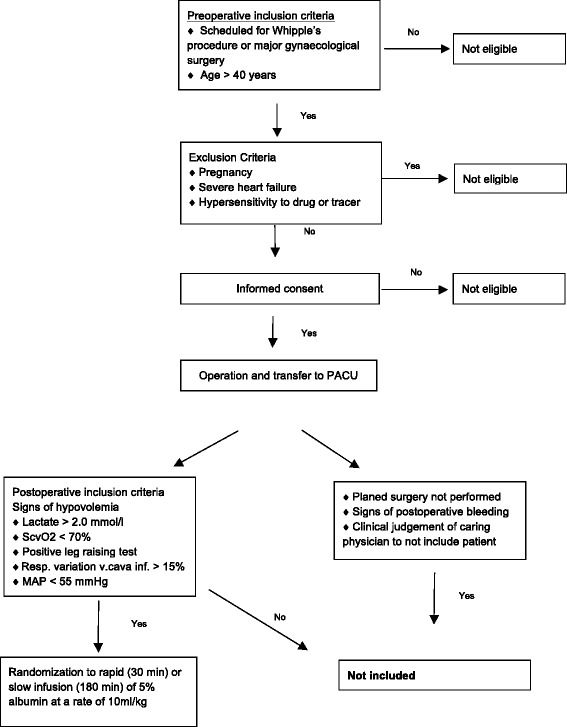
Detailed enrolment and randomisation flowchart for Albumin Infusion Rate (AIR) study. *PACU* Post-anaesthesia care unit, *ScvO*_*2*_ Central venous oxygen saturation, *MAP* Mean arterial pressure

We are conducting a single-centre, investigator-initiated, prospective, parallel-group, randomised study of two different albumin infusion rates in patients following major abdominal surgery. The study is approved by the regional ethical vetting board in Lund, Sweden (Dnr 2014/15), and will be conducted at Skåne University Hospital in Lund. Overviews of the trial design according to the Standard Protocol Items: Recommendations for Interventional Trials (SPIRIT) statement and an overview of patient flow through the study are presented in Table 1 and Fig. 1, respectively. A SPIRIT checklist is provided in Additional file 1. Informed consent will be obtained non-consecutively from eligible participants when a member of the research team is available. The length of the study will be from signing of informed consent until 36 ± 7 days after the start of infusion of the test substance. All patients will be followed by a study investigator in a visit at the trial centre. All patients will be evaluated with regard to potential adverse effects by one of the investigators. The first patient was included on 18 June 2014. The protocol has been amended two times. The first amendment extended inclusion criteria to also include post-operative patients after major gynaecological surgery to promote recruitment. The second amendment changed the interim analysis plan by adding the Haybittle-Peto approach for the testing of efficacy. In addition, ongoing vasopressor and inotropic therapy was omitted as an exclusion criterion. The first amendment was made after 1 patient had been included, and the second was made after 24 patients had been included. The amendments were approved by both the regional ethical vetting board and the Swedish Medical Products Agency (MPA), and the current protocol version 1.3 2015-04-03 was approved by MPA 2015-05-27.

**Table 1:** Overview of enrolment, interventions and assessments according to the SPIRIT (Standard Protocol Items: Recommendations for Interventional Trials) statement

	Study period							
	Enrolment	Allocation	Post-allocation					Close-out
Time point	-t_1_	0	t_1_	t_2_	t_3_	t_4_	t_5_	t_6_
Enrolment								
Eligibility screen	X							
Informed consent	X							
Allocation		X						
Interventions								
Infusion of 5% albumin (10 ml/kg) over 30 minutes				X				
Infusion of 5% albumin (10 ml/kg) over 180 minutes				X				
Assessments								
Plasma volume			X	X	X			
Transcapillary escape rate of albumin						X		
Haemodynamic data, haematocrit, lactate, ScvO_2_	X		X	X	X			
Post-operative complications			X	X	X	X	X	
Adverse events, serious adverse events			X	X	X	X	X	X

*Abbreviations: ScvO*_*2*_ Central venous oxygen saturation, *t*_*1*_ Baseline, *t*_*2*_ Start of albumin infusion, *t*_*3*_ 180 minutes after start of albumin infusion, *t*_*4*_ 180–240 minutes after start of albumin infusion, *t*_*5*_ 30 days after operation, *t*_*6*_ 36 ± 7 days after inclusion or until resolution of any adverse event, serious adverse event or suspected unexpected serious adverse reaction

### Inclusion criteria

Post-operative patients following non-emergent operation ad modum Whipple procedure or major gynaecological cancer surgery at the age of 40 years or older will be included if fulfilling the following criteria:

1. Indication for fluid therapy as judged by the physician caring for the patient and at least one of the following criteria is fulfilled within 5 h after admission to the post-anaesthesia care unit (PACU):
  - Positive ‘leg-raising test’ (pulse pressure increase >9% or stroke volume increase by >10% as measured with cardiac ultrasound [14]
  - Central venous oxygen saturation (ScvO_2_) <70%
  - Plasma lactate >2.0 mmol/L
  - Urine output <0.5 ml/kg in the hour prior to inclusion
  - Respiratory variation of the inferior vena cava >15% as measured by ultrasound [15, 16]
  - Systolic blood pressure <100 mmHg or mean arterial blood pressure <55 mmHg
2. Written consent by the patient to participate in the study obtained prior to operation

### Exclusion criteria

Patients fulfilling any of the following criteria will be excluded from the study:

1. Hypersensitivity to the active drug or the tracer
2. Signs of post-operative bleeding
3. History of heart failure
4. The physician caring for the patient considers that there are strong reasons to administer another fluid, or the same fluid but in another way or in a different volume than stated in the protocol
5. Pregnancy
6. Clinical judgement by the investigator or the treating physician that the patient should not participate in the study for reasons other than described above

### Informed consent and withdrawal

Patients scheduled for the operative procedures described above will be assessed for inclusion. If none of the pre-operative exclusion criteria are met, a member of the research team will provide both oral and written information before surgery (Additional file 2). All patients will be given the opportunity to ask questions about the study and will also be given sufficient time to decide whether to participate. Patients are informed of their right to withdraw from the study at any time. A patient who withdraws consent receives standard care. In addition, the patient/participant may be permanently withdrawn at the investigator’s or the treating physician’s discretion at any time if this is considered to be in the patient’s best interest. Predefined permanent withdrawal criteria are as follows:

1. Change of surgery procedure to other than operation ad modum Whipple or major gynaecological cancer surgery
2. Serious violation of the study protocol, such as administration of other resuscitation fluid in a volume >200 ml within 3 h after start of albumin infusion
3. Occurrence of known side effects, defined as serious adverse events (SAEs) listed in the summary of product characteristics of 5% albumin or SERALB-125® (IBA-CIS Bio International, Gif-Sur-Yvette, France) (Side effects that warrant permanent withdrawal include anaphylactic shock or pulmonary oedema.)

A record is kept of patients who consented in the pre-operative screening but were never enrolled (patient screening log).

### Intra- and post-operative care of the patients

Eligible patients who have given consent to participate in the study will receive routine pre- and intra-operative care. Anaesthesia will be induced intravenously using propofol and maintained using either sevoflurane or desflurane. Patients will receive an epidural catheter for intra- and post-operative analgesia unless contraindicated. Fentanyl will be used as an analgesic during induction, and rocuronium will be used as a muscle relaxant. Crystalloids and colloids will be used as resuscitation fluids intra-operatively at the discretion of the attending anaesthetist. A haemoglobin level of 80–90 g/L will be the transfusion trigger. Post-operative maintenance fluids will be a 2.5% or 5% glucose solution at a rate of 1 ml/kg/h. Analgesics may be given during the intervention phase of the study, and the rate of vasoactive agents may be adjusted to maintain mean arterial pressure >65 mmHg. No fluid other than maintenance and study fluids may be given during the intervention period.

### Randomisation and blinding

Patients are screened for indications for fluid administration during the first 5 h after admission to the PACU as described above. Patients who fulfil inclusion criteria and meet no exclusion criteria will be randomised using a sealed envelope to either rapid or slow infusion of study drug. The randomisation and preparation of sealed envelopes are performed by an independent party (Clinical Research Unit, Skåne University Hospital, Lund). Randomisation was performed using a computerised random number generator. The research team is blinded to block size. Measurements of plasma volumes, transcapillary escape rate (TER) for albumin and post-operative follow-up will be performed by members of the research team who are blinded to the treatment allocation.

### Study interventions

**Fig. 2:**
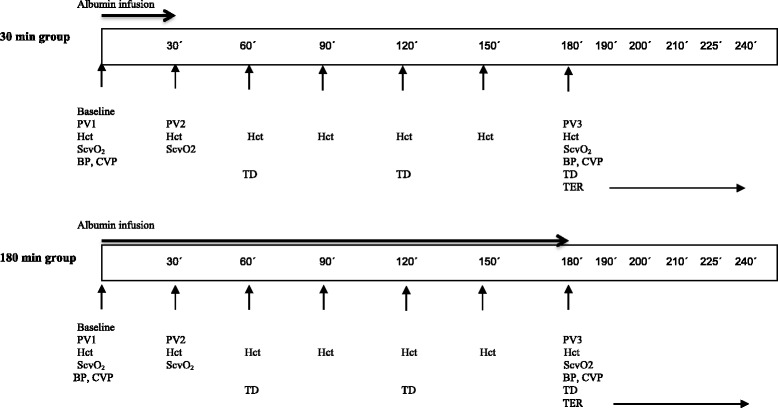
Detailed experimental protocol. *PV1* Baseline plasma volume, *PV2* Plasma volume after 30 minutes, *PV3* Plasma volume after 180 minutes, *Hct* Haematocrit, *ScvO*_*2*_ Central venous oxygen saturation, *BP* Blood pressure, *CVP* Central venous pressure, *HD* Hourly diuresis, *TER* Transcapillary escape rate

Patients randomised to a fast or slow infusion will receive 5% albumin at a dose of 10 ml/kg in 30 minutes or 180 minutes, respectively. Dose is based on ideal body weight [17]. At 240 minutes after the start of albumin infusion, the study protocol is completed, and the patient thereafter receives post-operative care according to local routine. Figure 2 shows a brief overview of the experimental protocol. Patients will be in lying position throughout study protocol time (240 minutes).

### Primary outcome

The primary outcome in the study is change in plasma volume 180 minutes after the start of the albumin infusion.

### Secondary outcomes

Secondary outcomes are differences in plasma volume over time (integral of plasma volume over time from the start of albumin infusion from plasma volume PV1 to PV3) and incidence of post-operative complications up to 30 days post-operatively (see Additional file 3 for definitions of complications). Other outcomes of interest are TER for albumin from 180–240 minutes after the start of albumin infusion, change in heart rate, change in central venous oxygen saturation, change in haemoglobin concentration in blood, change in blood pressure, change in central venous pressure, change in plasma lactate from the start of albumin infusion to 3 h after start of infusion, and diuresis from the start of albumin infusion to 3 h after the start of infusion.

In addition, exploratory analyses are planned. These analyses include measurement of effects of the different infusion rates on plasma concentration of markers of endothelial damage and on hormones involved in fluid homeostasis. For this purpose, blood samples are collected 5 minutes before centrifugation, and plasma samples will be stored following centrifugation at −80 °C until analysis. We also intend to analyse to what extent included patients are hypovolaemic at inclusion (>10% lower than calculated normal blood volume [18]).

### Measurements

Plasma volume is measured using ^125^I human serum albumin (HSA) (SERALB-125®) in a total dose of at most 0.4 MBq. The dose administered to each patient is recorded in the case report form (CRF, Additional file 4). The dose in the syringes is determined from their increased weight multiplied by an activity concentration from a standard. This standard was prepared from a small sample from the ^125^I-HSA vial added to a test tube and measured for both activity and weight. Blood samples are collected 5 minutes prior to injection of ^125^I-HSA and 10 minutes after injection of ^125^I-HSA for the plasma volume calculations. Plasma concentration is determined in a gamma counter (PerkinElmer 1480 Wizard; PerkinElmer, Waltham, MA, USA), and plasma volume is calculated by dividing the injected dose of ^125^I-HSA by the change in concentration of ^125^I-HSA in plasma at 10 minutes post-injection. Injected doses are corrected for remaining activity in the syringes.

Haematocrit is measured by colorimetric analysis using a blood gas analyser (Radiometer 850; Radiometer, Copenhagen, Denmark). Assuming that no blood loss occurs during the study period, changes in haematocrit will reflect changes in plasma volume. By this methodology, plasma volumes can be estimated every half-hour during the infusion of albumin. By measuring plasma volume with ^125^I-HSA at three different points in time, the potential effect of alterations in small- to large-vessel haematocrit can be corrected [19]. The area under the plasma volume curve will then be calculated for each patient (plasma volume over time).

The TER for albumin is a measure of leakage of albumin from microvessels into the microvasculature. Changes in TER reflect changes in microvascular permeability and changes in parameters influencing convective transport of albumin such as transvascular hydrostatic pressure. Plasma concentration of ^125^I-HSA is measured at 10 minutes, 30 minutes, 45 minutes and 60 minutes after the last injection of the tracer. Plasma concentration of ^125^I-HSA as a function of time will be plotted in a log-linear plot. The slope of the line represents TER and will be expressed as the percentage decrease in plasma concentration of ^125^I-HSA per hour [20–22]. For a summary of measurements, please see Fig. 2.

### Data collection and management

All study data will be recorded in CRFs for each patient, which are kept at the study site. Information on co-morbidities, medications, results of pre-operative physical examinations, routine laboratory analysis results, American Society of Anesthesiologists physical status classifications, Physiological and Operative Severity Score for the enUmeration of Mortality and morbidity and Revised Cardiac Risk Index will be collected from the hospital electronic chart system. Peri-operative data will be collected from the anaesthesia chart, and data that will be registered include anaesthesia methods and drugs, type and volume of fluids, peri-operative bleeding, and diuresis. Inclusion criteria will be registered in CRFs before randomisation. Clinical haemodynamic evaluation will be performed and recorded at inclusion and at 180 minutes after the start of albumin infusion. Members of the research team have unlimited access to study data. The data collected in the present study will be available from the corresponding author on reasonable request. The auditing (see below) will include source data verification.

### Sample size

Published SD values for plasma volume measured with the ^125^I-HSA method are in the range of 4–7 ml/kg [22]. In the present study, we wish to compare differences in changes in plasma volume, and it is reasonable to assume that the SD is in the lower part of this range. In an experimental study, a 50% greater plasma volume expansion was found after slow administration of 5% albumin compared with a bolus dose of the same volume [10]. If we wish to detect a 40% difference in volume-expanding effect following administration of 10 ml/kg of 5% albumin, assuming that the SD of the plasma volume is 5, then about 30 patients in each group are required to obtain a power of 80%. To adjust for a slightly lower than expected treatment effect and for the possibility that patients may not complete the protocol, we intend to include a total of 70 patients: 35 patients in each arm. Should the number of patients who complete the protocol be <30 in any of the treatment groups, we plan to increase the sample size to maintain power.

### Statistical analysis plan

The study will continue until the planned number of patients has been included, unless the interim analysis indicates that the study should be stopped. Results will be unblinded when all data have been collected. Only patients who received 90% or more of the intended dose will be included in the analysis. The investigating team will perform the statistical analyses.

#### General analytical principles

1. Analysis will be performed on a per-protocol basis.
2. All hypothesis tests will be two-sided, with a maximal type I error risk of 0.05.
3. Subgroup analysis will be performed regardless of overall treatment efficacy.
4. Imputation will not be used to correct for missing data in the analysis.

#### Assessment of baseline variables

Baseline variables of patients fulfilling study protocol in the two study arms will be tabulated. Discrete variables will be reported as frequencies and percentages, and continuous variables will be reported as either means with SDs or medians with interquartile ranges.

#### Analysis of outcomes

The primary outcome will be analysed using Student’s *t* test. Secondary outcomes will be analysed using Student’s *t* test or the Mann-Whitney *U* test as appropriate. Differences between the groups will be reported as means with 95% confidence intervals. In the event of no difference between the groups with regard to primary outcome and a difference in the secondary outcome of plasma volume over time, we will interpret these results as supporting our hypothesis but that confirmatory studies are needed. With the exception of number of complications, all other outcomes are regarded as exploratory, and no emphasis will be placed on differences between the treatment groups, should the primary outcome and plasma volume over time outcome be negative.

#### Subgroup analysis

A sensitivity analysis using multivariate regression will be performed to assess if treatment effect is dependent on type of surgery and baseline plasma volume.

### Auditing

The purpose of auditing and quality control is to ensure scientific integrity, data quality, the safety and integrity of the participating subjects, and that the study is compliant with the current versions of the Declaration of Helsinki, the International Council for Harmonisation of Technical Requirements for Pharmaceuticals for Human Use, good clinical practice and national regulations. The sponsor will delegate auditing to the Clinical Research Unit, Skåne University Hospital in Lund, an independent party that will perform on-site monitoring before, during and after the study. Considering that the patients will be treated according to normal routine during major abdominal surgery, that the infusion rates and volumes are within the normal range, and the low frequency of adverse events (AEs) in an earlier large prospective randomised clinical trial comparing albumin with normal saline in an ICU setting, the study will be performed without the use of a data monitoring board [23].

### Interim analysis

An independent statistician will perform an interim analysis for assessment of efficacy and futility after 36 patients have completed the protocol. The Haybittle-Peto approach will be used when testing for efficacy. If a difference with regard to the primary endpoint with *p* ≤ 0.001 is detected, the study may be stopped. Futility will be assessed by simulating the remainder of the study multiple times using an SD of 5 and a difference in means of 4 ml/kg between the two groups. The results of each simulation will be combined with the obtained data. If the simulated data in combination with the observed data show a significant effect (two-sided Student’s *t* test with an α <0.05) in less than 10% of the cases, the study will be stopped. Should the observed SD in interim analysis be higher than that used in the power calculation, the higher number will be used for the simulation. The principal investigator has the authority to stop the trial.

### Harms

All patients are evaluated with regard to potential AEs or SAEs by one of the investigators, and potential AEs and SAEs are recorded in the CRF. A SAE is defined as an event that fulfils one or more of the following criteria: results in death, is life-threatening, requires prolongation of hospitalisation, results in persistent or significant disability or incapacity or any other important medical event. The investigator evaluating the patient at the end of the study is responsible for the treatment of any AE or SAE until resolution of the AE or SAE. Depending on the nature of the AE or SAE, the treatment will take place at either the trial centre, the local hospital or as an outpatient. If the responsible investigator/sponsor judges the SAE as being drug-related and unexpected, this must be promptly reported to the sponsor, which is responsible for reporting suspected unexpected serious adverse reactions to the Swedish MPA and the regional ethical vetting board.

### Publication plan

The study is registered in the European Clinical Trials Database (EudraCT 2013-004446-42) and the ClinicalTrials.gov database (NCT02728921). Following completion of the trial, the main manuscript will be submitted to a peer-reviewed journal, regardless of the trial outcome. For publication of the main outcomes, the first figure presented will be a Consolidated Standards of Reporting Trials (CONSORT) flowchart. The diagram will include the number of screened patients, the number of patients giving consent, the number of patients meeting all inclusion criteria, the number of patients randomised to each of the two treatment arms, and the number of patients completing the protocol in each of the treatment groups. The second figure will depict plasma volumes in the respective groups at baseline and at 30 minutes and 180 minutes after the start of albumin infusion. The first table will describe baseline demographics as detailed above. The second table will describe secondary outcomes. Authorship will be granted according to the criteria described by the International Committee of Medical Journal Editors [24].

### Funding and sponsor

The AIR trial is funded by Region Skåne (Medical Training and Research Agreement [Avtal om Läkarutbildning och Forskning]), the Gyllenstiernska Krapperup Foundation and the Anna and Edwin Berger Foundation. Other than funding, the funders have no role in any aspect of the trial. The study principal investigator (PB) is also sponsor as delegated by Skåne University Hospital.

## Discussion

The AIR study will provide clinical data on the importance of infusion rate for the plasma volume-expanding effect of colloids in patients with suspected hypovolaemia after major abdominal surgery. The decision to include patients subjected to a Whipple procedure or major gynaecological surgery is based on the clinical experience that these patients often appear hypovolaemic in the immediate post-operative period and thus are likely to require fluid resuscitation. Post-operative hypovolaemia in these patients is at least partly due to vascular leak secondary to the systemic inflammatory response initiated by the operation, and these patients are therefore likely to share pathophysiological mechanisms with other critically ill patients requiring fluid resuscitation [25]. Given the transient nature of the need for fluid resuscitation in the majority of these post-operative patients, the immediate post-operative period was believed to represent the optimal time point for our purposes.

Albumin is chosen as the study colloid because albumin is a naturally occurring colloid with very few contraindications in the current setting and with an excellent safety record [23]. The rates of infusion are chosen to differ as much as possible within the range of clinical practice for this patient category. In routine clinical practice, the anaesthesiologist caring for the patient decides how quickly 5% albumin is to be administered, and the rates and doses to be tested are within the limits normally used in our institution as well as in others, and they do not differ from our clinical routine [26, 27].

Plasma volume measurement using ^125^I-HSA is considered to be the gold standard for measurement of plasma volume and is used clinically in many hospitals worldwide. The radiation dose received by the patients due to participation in the study is approximately 0.12 mSv and is less than the background radiation that patients are naturally exposed to during a 6-month period, and also <0.1% of the dose used during radioiodine treatment of hyperthyroidism. Therefore, in accordance with World Health Organisation guidelines, patients will not receive treatment with potassium iodide to block thyroid uptake of radiolabelled iodine.

Measurement of TER for albumin is commonly used to evaluate vascular leak of albumin. After a bolus dose of ^125^I-has, there is a steep decline in plasma concentration during the first 10 minutes, which is thought to represent mixing and distribution into a rapidly equilibrating space. After the first 10 minutes, the rate of decline in plasma concentration of ^125^I-HSA reflects extravasation and a gradual increase in lymphatic return of the tracer. To minimize the influence of lymphatic return on the decrease in in plasma concentration of tracer, TER is measured during the first hour after injection. It could be argued that recirculation of ^125^I-HSA from the two preceding plasma volume measurements (see Fig. 1) could influence the TER measurement. The magnitude of this influence during the 4-h experimental period can be modelled by assuming a TER value of 15%, a ratio of 1:4 between the intravascular and interstitial extracellular compartments. On the basis of these assumptions, it can be estimated that recirculation will have a negligible influence on a difference in TER between the two treatment groups (Additional file 5). This conclusion aligns with results from a similar analysis concerning repeated TER measurements in patients with sepsis [28].

### Trial status

Recruitment has been completed. The interim analysis has been performed, and no reason to halt the study has been found. No SAEs have been registered to date.

## Additional files

Additional file 1: SPIRIT Checklist. (DOC 99 kb)
Additional file 2: Consent form (in Swedish). (DOCX 32 kb)
Additional file 3: Definition of postoperative complications. (PDF 108 kb)
Additional file 4: Case report form (in Swedish). (DOCX 258 kb)
Additional file 5: Effect of tracer recirculation on measuremnet of transcapillary escape rate for albumin. (DOCX 158 kb)

## Abbreviations

AE: Adverse event
AIR: Albumin Infusion Rate study
BP: Blood pressure
CONSORT: Consolidated Standards of Reporting Trials
CRF: Case report form
CVP: Central venous pressure
Hct: Haematocrit
HD: Hourly diuresis
HSA: Human serum albumin
ICU: Intensive care unit
MAP: Mean arterial pressure
MPA: Swedish Medical Products Agency
PACU: Post-anaesthesia care unit
PV: Plasma volume
SAE: Serious adverse event
ScvO_2_: Central venous oxygen saturation
SIRS: Systemic inflammatory response syndrome
SPIRIT: Standard Protocol Items: Recommendations for Interventional Trials
TER: Transcapillary escape rate
