# Supplementary material for: The importance of albumin infusion rate for plasma volume expansion following major abdominal surgery – AIR: study protocol for a randomised controlled trial
Source: Trials. 2016 Dec 7;17:578. doi: 10.1186/s13063-016-1714-5 (PMC5142270; doi:10.1186/s13063-016-1714-5)
Supplement: Additional file 2: — Consent form (in Swedish). (DOCX 32 kb) [file 13063_2016_1714_MOESM2_ESM.docx]

**Patientinformation**

AIR-studien

**Har dropphastigheten någon betydelse för blodvolymsökande effekten av albumin i samband med operation?**

Du tillfrågas om att delta i en klinisk forskningsstudie då du skall genomgå en operation då man tar bort en del av bukspottskörteln och andra intilliggande organ eller genomgå gynekologisk cancerkirurgi.

På Skånes universitetssjukhus i Lund bedrivs patientnära forskning som syftar till att förbättra vården till patienterna. Studien bedrivs av kliniken för intensiv och perioperativ vård i samarbete med kirurgiska kliniken. Denna information beskriver hur studien ska genomföras och varför. Den beskriver också hur du går tillväga om du vill ta del av dina insamlade uppgifter. Ta god tid på dig att läsa informationen och ställ frågor om du undrar över något.

**Bakgrund och syfte**

För att säkerställa optimal funktion av kroppens organ behöver hjärtat pumpa en viss volym blod varje minut. I samband med operation så minskar blodvolymen vilket kan leda till för lågt blodflöde till olika organ. Alla patienter behandlas därför med vätska. Många vätskor som används läcker snabbt ut ur blodbanan och kan därigenom försämra olika organs funktion och det är därför av stor vikt att så mycket som möjligt av den vätska som tillförs stannar i blodbanan. En av de vätskor som används är en albumininnehållande saltlösning som används för att man tror att den stannar kvar i blodet längre än saltlösningar som inte innehåller albumin. Denna studie syftar till att ta reda på om dropphastigheten spelar någon roll för hur länge en albuminlösning stannar kvar i blodet. Om vi finner att det är en skillnad i dropphastigheten så skulle detta kunna leda till att patienter får en bättre organfunktion efter stora operationer och en kortare sjukhusvistelse.

**Vad innebär studien för dig/studieupplägg**

Studien är i alla sina delar granskad och godkänd av Regionala etikprövningsnämnden i Lund och Läkemedelsverket. Patienter som kommer till Skånes Universitetssjukhus för den sortens operation som du själv skall genomgå har möjlighet att delta. Om du väljer att delta innebär det följande: om du behöver vätska efter operationen kommer du att slumpmässigt lottas till att få denna vätska på antingen 30 minuter eller 3 timmar. Innan du får vätskan och efter att du fått den så kommer vi att mäta hur mycket vätska du har i blodet och koncentrationer av hormoner som har med vätskebalans att göra. För att kunna göra detta kommer vi att vid tre tillfällen injicera en liten mängd albumin som märks med en radioaktivt jod. Under 4 timmar efter att du fått albumin kommer vi att ta en del extra blodprover för att följa din blodvolym och hormonnivåer. I övrigt är vården identisk med den vård du får om du väljer att inte delta i studien. Vi kommer ca 1 månad efter att du skrivits ut från sjukhus att följa upp hur du mår, i samband med ett uppföljningsbesök som följer normal rutin. Därefter avslutas din medverkan i studien.

**Fördelar med att delta**

Du kommer inte att ha någon egen direkt nytta av att vara med i studien, För alla som deltar i studien kommer blodvolymen och blodkoncentrationen att följas noga. Dessa värden kommer förhoppningsvis på sikt att ge svar på om det är bättre att ge droppet långsamt än med snabb hastighet. Detta kan i framtiden leda till att patienter som kommer att göra denna operation, kan få mindre komplikationer och kortare sjukhusvistelse.

**Nackdelar med att delta**

Fler blodprover kommer att tas jämfört med om du inte deltar. Blodprovstagningen kommer att ske via en tunn plastslang som du får insatt i ett blodkärl. Totalt tas 86 ml blod som del av studien, vilket kan jämföras med att en blodgivare lämnar 450 ml blod vid ett och samma tillfälle.

Albumin märkt med radiojod (isotop) kommer att ges vid tre tillfällen för att kunna utföra volymmätningar i blodet. Detta kommer att ge en sammanlagd stråldos som motsvarar mindre än ett halvt års naturlig bakgrundsstrålning och mindre än en trettiondel av den stråldos som man får i samband med en datortomografiundersökning av buken. Isotopen är inte giftig.

Allergi eller feberreaktioner av radiojodmärkt albumin finns beskrivna. Detta är mycket ovanligt och du är noggrant övervakad av erfaren personal om något sådant skulle ske.

**Merkostnader/ersättning**

Du får inget betalt eller kostnadsersättning för att delta i studien. Ingen deltagande vårdpersonal har några ekonomiska intressen i studien.

**Försäkringsskydd**

För dig som deltar i studie gäller Patientskadeförsäkringen och Läkemedelsförsäkringen, på samma sätt som vid all annan behandling inom sjukvården*.*

**Antal patienter**

Totalt 70 patienter planeras ingå i studien här i Lund.

**Hantering av studiedata (sekretess)/insamling av personuppgifter**

All information som samlas in under din sjukhusvistelse och under studietiden

hanteras i enlighet med offentlighets- och sekretesslagen och personuppgiftslagen (PuL). Region Skåne är personuppgiftsansvarig för denna personuppgiftsbehandling och har godkänt insamlandet av data. Patientuppgifter som samlas in i studien såsom aktuell sjukhusvistelse och labdata kommer att lagras i ett register och databehandlas. Dina uppgifter är sekretesskyddade och ingen obehörig har tillgång till registret. I dataregistret och vid databearbetning kommer ditt namn att ersättas med en kod så att en enskild individ inte kan urskiljas. Endast den som är ansvarig för studien har kodnyckeln. Uppgifterna kommer under studiens gång att behandlas konfidentiellt och dina personuppgifter kommer att vara kodade och kodnyckeln förvars säkert för obehöriga utanför forskargruppen. Den insamlade informationen kan därför endast härledas till dig av ansvariga i forskningsprojektet. Studieresultat som rapporteras i vetenskapliga publikationer redovisas endast på gruppnivå och din identitet kommer inte att avslöjas

En oberoende person kommer att ha tillgång till din patientjournal för att granska studiens utförande och för att säkerhetsställa att datainsamlingen sker på ett korrekt sätt och att dina intressen som patient har tillgodosetts. Detta kommer naturligtvis att ske under förbehåll av sekretess. Ingen information kommer att bli tillgänglig eller spridas till andra.

Enligt personuppgiftslagen har du rätt att ansöka om information/registerutdrag en gång/år om de uppgifter som samlas in om dig. Detta gör du genom att skriva till Personuppgiftsombudet, Region Skåne, 291 89 Kristianstad. Ansökan måste vara egenhändigt undertecknad. Du har också rätt att få eventuellt felaktiga personuppgifter rättade.

**Hantering av blodprover**

Blodprover kommer att analyseras på Skånes Universitets sjukhus och på laboratorier utanför regionen. Blodproven kommer att tillhöra en biobank hos Region Skåne. En

biobank innehåller biologiskt material från människor som lagras under en längre tid, där de enskilda proverna går att koppla till de personer som de kommit ifrån.

Blodproven hanteras enligt samma rutiner som finns för sjukvårdens prover. Proverna

förses med en kod, som inte innehåller persondata. Detta innebär att inga

uppgifter om din identitet såsom namn, ålder eller födelsedatum kommer att finnas på

ditt blodprov. Kodnyckel förvaras hos ansvarig läkare.

Proverna kommer endast att användas för de ändamål som angivits och kan endast bli aktuella för ett nytt ändamål efter ny prövning hos etikprövningsnämnd. Du har rätt att

och utan närmare förklaring begära att dina prover ska förstöras.

**Frivillighet**

Deltagande i studien är helt frivilligt och du kan när som helst avbryta deltagandet utan att ange något skäl. Ditt fortsatta omhändertagande kommer inte att påverkas i den fortsatta behandlingen. Om någon ny information om studieläkemedlet blir tillgänglig som kan påverka ditt beslut att fortsätta delta kommer du att bli informerad om detta. Din läkare har också rätt att avbryta ditt deltagande i studien om ny information framkommer som gör det lämpligast för dig att avbryta studien.

**Kontaktperson**Ansvarig för studien är Dr Peter Bentzer, Docent och specialistläkare Anestesi och Intensivvård, Skånes Universitetssjukhus i Lund. +46 46 172970, +46 70 7197372, [peter.bentzer@hotmail.com](mailto:peter.bentzer@hotmail.com)

**Samtycke till deltagande i studien**

Jag har informerats muntligen om studien och har tagit del av ovanstående skriftliga information och fått svar på mina frågor och samtycker till att delta.

- Jag är medveten om att deltagande i studien är fullt frivilligt och att det när som helst och utan närmare förklaring går att avbryta detta deltagande utan att det påverkar mitt framtida omhändertagande.

*-* Jag har fått information om att de uppgifter som samlats in om mig i studien kommer att behandlas enligt personuppgiftslagen. Mitt samtycke gäller under förutsättning att detta sker konfidentiellt, vilket innebär att min identitet inte kommer att röjas för obehöriga.

- Jag tillåter att en av studieledningen utsedd oberoende person och/eller myndighetsperson får jämföra studieuppgifter med information i min medicinska journal. Detta får ske under förutsättning att sedvanlig sekretess upprätthålls.

- Jag samtycker till att de prover som tas i studien hanteras enligt biobankslagen. Jag är medveten om att mitt provmaterial inte kommer att användas till annat syfte än de ovan nämnda utan att jag informerats och gett nytt samtycke till detta, samt att regional etikprövningsnämnd har lämnat sitt godkännande.

Jag är informerad om rättigheten att få registerutdrag en gång per år över vilka uppgifter och eventuellt provmaterial som är insamlat. Jag har dessutom informerats om rättigheten att få provmaterial förstört eller avidentifierat och att få felaktiga uppgifter tillrättalagda eller borttagna ur registret.

*___________________________________________________________________________*

Ort och datum Patientens namnteckning

*………………………………………………*

Namnförtydligande

Jag har givit denna patient/anhörig muntlig och skriftlig information om studien Jag har svarat på frågor om studien och har övertygat mig om att patienten har förstått. Jag har därefter inhämtat det skriftliga medgivandet att deltaga i studien. En kopia av den skriftliga patientinformationen och det skrivna samtycket överlämnas till patient/ anhörig.

…………………………………………………………………………………………………...

Ort och datum Informerande läkares namnteckning

…………………………………………

Namnförtydligande
