# Supplementary material for: The importance of albumin infusion rate for plasma volume expansion following major abdominal surgery – AIR: study protocol for a randomised controlled trial
Source: Trials. 2016 Dec 7;17:578. doi: 10.1186/s13063-016-1714-5 (PMC5142270; doi:10.1186/s13063-016-1714-5)
Supplement: Additional file 3: — Definition of postoperative complications. (PDF 108 kb) [file 13063_2016_1714_MOESM3_ESM.pdf]

Appedix A. Definition of complications used as secondary outcomes.

**Infectious:**	
Pneumonia	X-ray signs + clinical signs or increase in CRP/temperature + treatment with antibiotics
Abdominal infection	Clinical signs + increase in CRP/temperature (>38.5° C) + treatment with treatment with antibiotics
Wound infection	Clinical signs (rubor, calor, dolor, functio laesa) + increase in CRP/temperature or positive culture + treatment with antibiotics
Urinary tract infection	Leucocytes + nitrate on urine sticks or positive culture + treatment with antibiotics
Catheter infection	Local irritation + Clinical signs or positive culture + treatment with antibiotics
Sepsis	2 out of 4 SIRS criteria + likely infection + treatment with antibiotics
Septic shock	Sepsis necessitating inotropic support
Infection with unclear focus	CRP rise + fever + treatment with antibiotics
**Cardiovascular:**	
Myocardial infarction	1. Increase in Trop T + one of the following: typical symptoms for at least 15min or new infarction signs on ECG (Q-wave in at least 2 leads, new LBBB, new ST-T changes) or loss of viable myocardium as judged by new movement anomaly on cardiac ultrasound.
Postoperative hypotension	Mean arterial pressure <65 despite adequate volume transfusion, necessitating inotropic/vasopressor support
New arrhythmia	New persistent arrhythmia on ECG necessitating treatment
Pulmonary oedema	Clinical signs + x-ray
Stroke	New neurological deficit and CT findings consistent with stroke
Pulmonary embolism	CT or scintigram
Deep vein thrombosis	Ultrasound or angiogram
**Respiratory:**	
Pleural effusion	X-ray or ultrasound
Pulmonary embolism	CT scan or lung scintigraphy
Prolonged need for respiratory support	Reintubation/NIV
Secretions necessitating interventions	Clinical signs + intervention (deep suctioning, extra physiotherapy, NIV, intubation)
ALI/ARDS	Sudden onset + bilateral infiltrates on x-ray (in absence of left heart failure) + PaO2/FiO2 < 300/200
**Abdominal:**	
Prolonged paralytic ileus	No bowel movement > 6 days postoperatively
Intraabdominal hypertension	>20 cmH_2O surgical intervention necessary
Abscess	x-ray + clinical signs
Intestinal ischemia	Visual diagnosis during reoperation
Anastomotic leakage	Visual diagnosis during reoperation
Wound dehiscence	Surgical intervention necessary (in the ward or in theatre)
**Renal:**	
Acute Kidney Injury	According to the KDIGO practice guideliner Kidney Inter. Suppl 2012
Need for dialysis	Dialysis
**Bleeding disorders:**	
Gastrointestinal bleeding	Clinical signs + pharmacologic or surgical intervention
Coagulopathy	PK>1.8 + APTT>60 sec or platelets count < 80.000/uL
Unspecified bleeding	Transfusion of >1 unit of erythrocytes postoperatively
**Prolongeded stay in PACU or admission to ICU**	Still in PACU at 10.00 a.m. the first postoperative day due to need for prolonged observation
