# Supplementary material for: The importance of albumin infusion rate for plasma volume expansion following major abdominal surgery – AIR: study protocol for a randomised controlled trial
Source: Trials. 2016 Dec 7;17:578. doi: 10.1186/s13063-016-1714-5 (PMC5142270; doi:10.1186/s13063-016-1714-5)
Supplement: Additional file 4: — Case report form (in Swedish). (DOCX 258 kb) [file 13063_2016_1714_MOESM4_ESM.docx]

**CASE REPORT FORM**

AIR

**THE IMPORTANCE OF ALBUMIN INFUSION RATE FOR**

**PLASMA VOLUME EXPANSION FOLLOWING MAJOR ABDOMINAL**

**SURGERY**

**Exklusionskriterier:**

🞏 graviditet

🞏 hjärtsvikt (NYHA klass 3 eller 4)

🞏 misstanke om pågående blödning

🞏 överkänslighet mot tracer eller mot studiedrog

🞏 patientansvarig anestesiolog anser att det finns starka skäl att behandla med en annan vätska eller samma vätska i en annan volym eller i en annan hastighet

🞏 patientansvarig anestesiolog eller medlem av forskningsgruppen anser att det finns andra kliniska skäl som gör att patienten inte bör vara med i studien.

**Inklusionskriterier:**

🞏 informerat samtycke

🞏 genomgången icke-akut stor gynekologisk eller stor bukoperation ad modum Whipple

🞏 ålder > 40 år

🞏 indikation för vätsketerapi och åtminstone 1 av nedastående vilkor är uppfyllda

🞏 positivt legraising test (pulstrycksökning > 9% eller slagvolymsökn > 10% mätt med UKG)

🞏 ScvO_2_ < 70%

🞏 laktat > 2mmol/l

🞏 urinproduktion < 0.5ml/kg/h senaste timmen

🞏 respiratorisk variation i v. cava inf dimameter > 15%

🞏 systolisk blodtryck < 100mmHg eller MAP < 55mmHg

**Bakgrundsdata Datum________________**

**ASA** _____________

**P-POSSUM**_____________

**Revised Cardiac Risk Index**__________________

**Längd________**

**Aktuell vikt__________**

**Ideal body weight ______________**

Man: 51 + 0.9 x (längd-152.9)

Kvinna: 45.5 + 0.91(längd-152.9)

## Födelsedag:

**År Mån Dag**

______________________

**Kön:** 🞎 Man 🞎 Kvinna

**Data för POSSUM och RCRI**

**(**[**http://www.riskprediction.org.uk/pp-index.php**](http://www.riskprediction.org.uk/pp-index.php)**)**

**(**[**http://www.mdcalc.com/revised-cardiac-risk-index-for-pre-operative-risk/**](http://www.mdcalc.com/revised-cardiac-risk-index-for-pre-operative-risk/)**)**

**­­­­Cerebrovaskulär sjukdom:**

Nej 🞎

**Ja 🞎** **○ TIA ○ stroke ○ annat: ________________________________________­­­__**

**Lungsjukdom:**

Nej 🞎

**Ja 🞎** **○ KOL ○ annat: ­__________________________**________________________

**Dyspne:**

Nej 🞎

**Ja 🞎**

| **○** | (No Dyspnoea) Ingen dyspne |
| --- | --- |
| **○** | (Dyspnoea on exertion) Dyspne vid ansträngning |
| **○** | (Limiting dyspnoea (on flight) moderate COAD) Begränsande dyspne, moderat KOL |
| **○** | (Dyspnoea at rest (rate > 30/min) fibrosis or consolidation) Dyspne i vila (RR >30/min) fibros eller konsolidation |

**Insulin behandlad diabetes:**

Nej 🞎

Ja 🞎______________________________

**Hjärtsvikt:**

Nej 🞎

**Ja 🞎** **○** **perifera ödem ○ hjärtförstoring ○ sänkt EF**___________________________

**Ischemisk hjärtsjukdom:**

Nej 🞎

**Ja 🞎** **○ MI<6mån ○ MI>6mån ○ angina**

**Malignitet:**

Nej 🞎

**Ja 🞎** ___________________________________________________________________

**Status vid inskrivning:**

Systoliskt blodtryck_______________

Hjärtfrekvens____________________

Andningsfrekvens________________

GCS______ RLS _______Temp_______

Rtg pulm patologi: Nej 🞎 Ja 🞎 **○ inkompensation ○ hjärtförstorning ○ emfysem**

EKG patologi: Nej 🞎 Ja 🞎 **○ FF (frekvens 60-90) ○ Q-våg ○ ST förändringar**

Annat______________________________________________________________________

####

**Läkemedel**

**­­­­ Kommentar**

**β-blockad** Nej 🞎 Ja 🞎____________________________________

**ACE hämmare** Nej 🞎 Ja 🞎____________________________________

**AT II antagonister** Nej 🞎 Ja 🞎____________________________________

**Statin** Nej 🞎 Ja 🞎____________________________________

**Diuretika** Nej 🞎 Ja 🞎____________________________________

**Trombyl** Nej 🞎 Ja 🞎____________________________________

**Insulin** Nej 🞎 Ja 🞎____________________________________

**Perorala antidiabetika** Nej 🞎 Ja 🞎____________________________________

**Steroider** Nej 🞎 Ja 🞎____________________________________

**Inhalationer** Nej 🞎 Ja 🞎____________________________________

**Waran** Nej 🞎 Ja 🞎____________________________________

**Nitropreparat** Nej 🞎 Ja 🞎____________________________________

**Digoxin** Nej 🞎 Ja 🞎____________________________________

**Preop lab. data Datum**

| Hb Hct | Krea | Vita | Na |
| --- | --- | --- | --- |
| K | CRP | PK | APTT |
| Glukos | Urea | Trc | Laktat |

**Operationsinformation**

| Ringa in ͽ | Des | Iso | Sevo | Annat: |
| --- | --- | --- | --- | --- |
|  | Pento | Propofol |  | Annat: |
|  | Esmeron | Tracrium | Celo | Annat: |
|  | Opioid | Perfalgan | NSAID | Annat |
|  | Atropin | Betapred | Ondansetron |  |
|  | Dridol |  |  |  |

**Komplikationer:** ­­­­­­­­­­­­­­­­­­­­­­­­­_______________________________________________

**EDA** Ja 🞎 Nej 🞎 **aktiverad peropertiv:** Ja 🞎 Nej 🞎

**Blödning**  _________ ml

**Op tid _________** min

**Anestesi tid** ________­_ min

**Total intraop. diures ______**___­_ ml

**Vätsketillförsel intraoperativ (ml)**

Ringer

NaCl

Glucoslösning

Venofundin

Macrodex

SAG

Plasma

Albumin

Övrigt

**Vasoaktiva farmaka**

Noradrenalin JA 🞎 NEJ 🞎

Adrenalin JA 🞎 NEJ 🞎

Dobutrex JA 🞎 NEJ 🞎

Dopamin JA 🞎 NEJ 🞎

Phenylefrin JA 🞎 NEJ 🞎

Efedrin JA 🞎 NEJ 🞎

Annat

**Hemodynamiska och andra data vid inklusion**

*Tryck anges som medelvärde av 2-3 mätningar över 10 minuter*

Systolisktblodtryck:1______2______3______ medel_______

Medelartärtryck:1_____2______3______ medel_______

CVP: 1_____2______3_______ medel_______

Laktat:___________ScvO2____________Hjärtfrekvens__________

Andningfrekvens_________ Temp_________

Pulstryck vid kontroll:

 halvsittande 30 - 40 grader vinkel höft- torso: _____________

Pulstryck vid benlyft:

 ben lyfta i 30- 40 grader mot horisontell torso efter en minut: _____________

Timdiures närmaste hela timme innan inklusion:___________

**Arteriell blodgas:** FiO2_________

PO2:_______ PCO2________ BE________ pH________ Saturation_______ Ht_______

🞏 basal vätsketerapi 5% Glukos 40 mmol Na 20 mmol K/1000ml 1ml/kg/h

Andra tillsatser än ovan 🞏 Nej 🞏 Ja: vilka?____________________________________

🞏 baslinje prov för PV bestämning: EDTA 5 ml

🞏 baslinjeprov för renin, angiotensin, ADH, ANP, syndekan-1, trombomodulin (Li Hep 3ml och EDTA 5 ml)

**Randomisering**

🞎 **snabb (30 min)** 🞎 **långsam (180 min)**

**Beräknad albumin volym (10ml/kg x ideal body weight): __________ ml**

**Räknad hastighet på infusion**________________ **ml/h**

| **Baslinje** |  |  |  |  |  |  |  |  |  |  |  |  |  |  |  |  | |
| --- | --- | --- | --- | --- | --- | --- | --- | --- | --- | --- | --- | --- | --- | --- | --- | --- | --- |
| min | **0** | **10** | **T0** | **25** | **30** | **40** | **60** | **90** | **120** | **150** | **175** | **180** | **190** | **210** | **225** | **240** |  |
|  | ^125^-I HSA  PV nr. 1 |  |   **ALB** |  | ^125^-I HSA  PV  nr. 2 |  |  |  |  |  |  | ^125^-I HSA  PV nr. 3 |  |  |  |  |  |
|  |  |  | TD nollas |  |  |  | TD |  | TD |  |  | TD |  |  |  |  |  |
| ABG |  |  |  | ABG |  |  | ABG | ABG | ABG | ABG | ABG |  |  |  |  |  |  |
| VBG |  |  |  | VBG |  |  |  |  |  |  | VBG |  |  |  |  |  |  |
|  |  | EDTA Nr.1  kyld  5 ml |  | EDTA Nr. 2  Kyld  5 ml |  | EDTA  Nr. 3  Kyld  5 ml |  |  |  |  | EDTA Nr. 4  Kyld  5 ml |  | EDTA  Nr. 5  Kyld  5 ml | EDTA  Nr. 6 kyld  5 ml | EDTA  Nr. 7  Kyld  5 ml | EDTA  Nr. 8  Kyld  5 ml |  |
| EDTA rör 7 ml kyld |  |  |  | EDTA rör 7 ml kyld |  |  |  |  |  |  | EDTA  rör 7 ml kyld |  |  |  |  |  |  |
| EDTA rör 5 ml i rums temp |  |  |  | EDTA rör 5 ml i rums temp |  |  |  |  |  |  | EDTA rör 5 ml i rums temp |  |  |  |  |  |  |
| Hep  rör 3 ml kyld |  |  |  | Hep  Rör 3ml  kyld |  |  |  |  |  |  | Hep  rör 3ml  kyld |  |  |  |  |  |  |

**Flödes schema: Bocka av och signera efterhand som HSA injiceras och prover tas.**

OBS ! Om arteriell och venös gas tagits inom 30 min så behöver man inte ta om det.

Säkerställ att EDTA rör 5 ml rymmer 4.5 ml Blod. Det finns en modell som endast rymmer 4 ml.

**Hemodynamiska och andra data vid T0 + 180 min**

*Alla tryck anges som medelvärde av 2-3 mätningar över 5 minuter*

Systolisktblodtryck:1_______2______3______ medel_______

Medelartärtryck:1_______2________3________ medel_______

CVP:1_______2______ 3______ medel_______

Laktat:___________ScvO2____________Hjärtfrekvens___________

Andningfrekvens _________ Temp_________

Pulstryck vid kontroll

 halvsittande 30 - 40 grader vinkel höft- torso _____________

Pulstryck vid benlyft

 ben lyfta i 30- 40 grader mot horisontell torso efter en minut: _____________

Timdiures: timme T0-60_________ T60-120_________ T120-180______________

**Arteriell blodgas** FiO2_________

PO2:_______ PCO2________ BE________ pH________ Saturation_____ Hct______

**PV**

**PV 1**

Counts baseline (3 ml) _____________cpm

Counts i spruta 1 _____________cpm

Counts i tom spruta 1 _____________cpm

Counts i plasma 10 min efter inj spruta 1 _____________cpm

**PV 2**

Counts T0 + 28 min _____________cpm

Counts i spruta 2 _____________cpm

Counts i tom spruta 2 _____________cpm

Counts i plasma min T0 + 40 _____________cpm

**PV 3**

Counts T0 +178 min _____________cpm

Counts i spruta 2 _____________cpm

Counts i tom spruta 2 _____________cpm

Counts i plasma min T0 + 190 _____________cpm

**TER**

Counts i plasma T0 + 190 _____________cpm

Counts i plasma T0 + 200 _____________cpm

Counts i plasma T0 + 210 _____________cpm

Counts i plasma T0 + 225 _____________cpm

Counts i plasma T0 + 240 _____________cpm

**Annan medicinering än basal vätska och studiedrog från T0 – T240**

Medicin klockslag och volym

___________________________________________________________________________

___________________________________________________________________________

____________________________________________________________________________

____________________________________________________________________________

**Postoperativa komplikationer tom 30 dagar postoperativt se appendix A. (notera datum och vilken komplikation)**

________________________________________________________________________________________________________________________________________________________________________________________________________________________________________________________________________________________________________________________________________________________________________________________________________________________________________________________________________________________________________________________

**Adverse Events**

Datum ______

Start: kl._____

Stop: kl._____

**Beskrivning av händelsen**:_________________________________________________________

______________________________________________________________________________
______________________________________________________________________________

**Åtgärd**_______________________________________________________________________

(no action, temporary stopped, permanently stopped)

**Outcome**______________________________________________________________________
(recovered, recovered with sequele, ongoing)

**Allvarlighetsgrad** (se sida 13) mild 🞏 moderat 🞏 svår 🞏

**Kausalitet i förhållande till studie läkemedel:** sannolik🞏 möjlig🞏 osannolik🞏

Om allvarlig meddela omedelbart Peter Bentzer på [peter.bentzer@hotmail.com](mailto:peter.bentzer@hotmail.com) alt [peter.bentzer@med.lu.se](mailto:peter.bentzer@med.lu.se)

Vid frågor, kontaktas:

Anestesiolog:

Namn: _________________________ mobil:_______________________

Vid akut situation kontakta IVA jour: tel. 72784

**Adverse Events**

Datum ______

Start: kl._____

Stop: kl._____

**Beskrivning av händelsen**:_________________________________________________________

______________________________________________________________________________
______________________________________________________________________________

**Åtgärd**_______________________________________________________________________

(no action, temporary stopped, permanently stopped)

**Outcome**______________________________________________________________________
(recovered, recovered with sequele, ongoing)

**Allvarlighetsgrad** (se sida 13) mild 🞏 moderat 🞏 svår 🞏

**Kausalitet i förhållande till studie läkemedel:** sannolik🞏 möjlig🞏 osannolik🞏

Om allvarlig meddela omedelbart Peter Bentzer på [peter.bentzer@hotmail.com](mailto:peter.bentzer@hotmail.com) alt [peter.bentzer@med.lu.se](mailto:peter.bentzer@med.lu.se)

Vid frågor, kontaktas:

Anestesiolog:

Namn: _________________________ mobil:_______________________

Vid akut situation kontakta IVA jour: tel. 72784

**Adverse Events**

**B** Datum ______

Start: kl._____

Stop: kl._____

**Beskrivning av händelsen**:_________________________________________________________

______________________________________________________________________________
______________________________________________________________________________

**Åtgärd**_______________________________________________________________________

(no action, temporary stopped, permanently stopped)

**Outcome**______________________________________________________________________
(recovered, recovered with sequele, ongoing)

**Allvarlighetsgrad** (se sida 13) mild 🞏 moderat 🞏 svår 🞏

**Kausalitet i förhållande till studie läkemedel:** sannolik🞏 möjlig🞏 osannolik🞏

Om allvarlig meddela omedelbart Peter Bentzer på [peter.bentzer@hotmail.com](mailto:peter.bentzer@hotmail.com) alt [peter.bentzer@med.lu.se](mailto:peter.bentzer@med.lu.se)

Vid frågor, kontaktas:

Anestesiolog:

Namn: _________________________ mobil:_______________________

Vid akut situation kontakta IVA jour: tel. 72784

**Vid fler än tre adverse events tryck ut ny sida 11 och märk med randomiseringsnummer och patientintialer**

**“Avslut på studien”**

**Fyll i sidan då uppföljning avslutas i samband med återbesök**

Datum för sista kontakt : _ _ _ _-_ _-_ _

År dag

→ Fullföljt studie protokoll. ja □ nej □

Alla patienter bör följas upp beträffande AE, SAE oberoende om de genomfört hela protokollet

**Om inte - ange skäl nedan**:

□ Samtycke återtaget

Skäl till detta:_________________________________

□ ”Lost to follow up”

□ Död. **Rapportera till**

**Peter Bentzer på SAE formulär inom 24h på** [**peter.bentzer@hotmail.com**](mailto:peter.bentzer@hotmail.com) **eller peter.benzer@med.lu.se**

Dödsdatum : _ _ _ _ - _ _ - _ _

År m dag

Dödsorsak:

□ Postoperativa komplikationer

□ Andra skäl, (beskriv):________________________________________

Obduktion nej □ ja□ om ja bifoga rapport

**Ansvarig Prövare**

Jag bekräftar att informationen i detta CRF är korrekt överensstämmer med källdata.

Ansvarig forskares underskrift:

Namnförtydligande :

Datum: _ _ _ _ - _ _ - _ _

År m dag

**Definition Adverse events**

*An adverse event (AE) is any untoward medical occurrence in a patient administered a*

*pharmaceutical product and which does not necessarily have a causal relationship with the*

*study treatment. An adverse event can therefore be any unfavourable and unintended sign*

*(e.g., tachycardia, enlarged liver) or abnormal results of an investigation (e.g., laboratory*

*finding, ECG), or symptom (e.g., nausea, chest pain) or disease temporally associated with the*

*use of a medical (investigation product), whether or not related to the medicinal*

*(investigational) product.*

**Definition Serious Adverse Events**

*A serious adverse event (SAE) is an AE occurring during any part of the study that fulfils one*

*or more of the following criteria:*

* results in death,*

* is life-threatening,*

* requires hospitalisation or prolongation of existing inpatients’ hospitalisation,*

* results in persistent or significant disability or incapacity*

* other important medical events*

*All SAEs will be reported to the sponsor within 24 hours after the investigator/sponsor has*

*become aware of it.*

**Assessment of severity**

*All adverse events will be assed for severity by the investigator.*

*• Mild: Awareness of sign or symptom, but easily tolerated and cause no interference*

*with daily activities.*

*• Moderate: Discomfort enough to cause interference with daily activities.*

*• Severe: Incapacitating with inability to perform normal daily activities.*

**Causal relationship to study drug**

*The investigator will judge whether or not, in his/her opinion; the adverse event is associated with the study treatment.*

*Probably: An adverse event, which might be due to the use of the drug. The relationship in*

*time is suggestive. An alternative explanation is less likely, e.g. concomitant drug (s),*

*concomitant disease(s).*

*Possibly: An adverse event, which might be due to the use of the drug. An alternative*

*explanation, e.g. concomitant drug(s), concomitant disease(s), is inconclusive. The*

*relationship in time is reasonable; therefore, the causal relationship cannot be*

*excluded.*

*Unlikely: An adverse event for which an alternative explanation is more likely, e.g.*

*concomitant drug(s), concomitant disease(s), or the relationship in time suggests that a*

*causal relationship is unlikely.*

**Adverse Event reporting**

*Following major surgery all patients will experience AEs and SAEs as defined above. Based*

*on this, expected effects of surgery and anaesthesia will not be reported. Signs and symptoms,*

*which are associated with the normal postoperative care following a Whipple operation will*

*therefore only be reported as AEs if it is both:*

* ****Serious according to the definition.***

*** Is not an expected sign or symptom as judged by the investigator***

*The following variables will be recorded in the CRF for each AE; description, start and stop*

*date, severity, SAE or not, causality rating, action taken and outcome of the AE.*

**Recording of AE**

***Adverse events occurring from signing of the consent until end of study will be recorded in the CRF.***

**Suspected Unexpected Serious Adverse Reaction (SUSAR)**

*All serious adverse events (SAE) must be evaluated unexpected or drug related or not. The*

*definition of an unexpected adverse reaction is an adverse event, which has not been*

*documented or reported earlier. The reference document is the SPC.*

*If the responsible investigator/sponsor judges the SAE as being drug related and unexpected it*

*must be promptly reported to the sponsor, who is responsible*

**SUSAR reporting**

*All serious adverse events (SAE) will be evaluated unexpected or not, drug related or not. The*

*definition of an unexpected adverse reaction is an adverse event, which has not been*

*documented or reported earlier. The reference document is the SPC.*

*If the responsible investigator/sponsor judges the SAE as being drug related and unexpected it*

*will promptly reported to the sponsor who is responsible for reporting SUSARs on the*

*CIOMS form to the Medical Product Agency in Sweden who will then report to the*

*Eudravigilance database.*

*A suspected unexpected serious adverse reaction to study drug (SUSAR), which is fatal or life*

*threatening will be reported to the LV and regional ethical committee within 7 days after the*

*principal investigator has become aware of the incident. Updates to complete the report must*

*be sent to the authorities within a maximum of 15 days.*

*A suspected unexpected serious adverse reaction to study drug (SUSAR), which is not fatal or*

*life threatening must be reported to the LV and regional ethical committee within 15 days*

*after the principal investigator has become aware of the incident. Updates to complete the*

*report must be sent to the authorities as soon as possible.*
