# Supplementary material for: The importance of albumin infusion rate for plasma volume expansion following major abdominal surgery – AIR: study protocol for a randomised controlled trial
Source: Trials. 2016 Dec 7;17:578. doi: 10.1186/s13063-016-1714-5 (PMC5142270; doi:10.1186/s13063-016-1714-5)
Supplement: Additional file 5: — Effect of tracer recirculation on measuremnet of transcapillary escape rate for albumin. (DOCX 158 kb) [file 13063_2016_1714_MOESM5_ESM.docx]

**Error estimations of TER-measurement using a Two-compartment model**

The trans-capillary escape and re-circulation of the albumin tracer molecule between the plasma and the interstitial compartment was simulated using a 2-compartment model. The time-dependent changes in plasma concentration (C_p_) and interstitial concentration (C_i_) were given by

$\frac{dC_{p}}{dt}= -TER\cdot C_{p}+TRR\cdot C_{i}-FCR\cdot C_{p}$ (1)

$\frac{dC_{i}}{dt}= +TER\cdot C_{p}-TRR\cdot C_{i}-FCR\cdot C_{i}$ (2)

where TER (h^-1^) is the trans-capillary escape rate, TRR (h^-1^) the ‘re-circulation rate’ of tracer from the interstitial compartment (Ci) and FCR (h^-1^) is the fractional catabolic rate representing the systemic catabolism (~4%/day) of albumin [[2](#_ENREF_2)]. The above system of differential equations was solved using a computer algebra system (MAXIMA version 5.26.0) which yielded a bi-exponential expression for the plasma concentration

$C_{p}\left( t \right)= C_{0}\frac{TERe^{-(TER+TRR+FCR)\cdot t}+TRRe^{-FCR\cdot t}}{TER+TRR}$ (3)

Here C_0_ is the initial plasma concentration (dose/plasma volume). Bolus doses were simulated using a simple step function

$H\left( t \right)=\frac{1}{1+e^{-8t}}$ (4)

The protocol was simulated as three repated bolus doses given at -15 min, +25 and +180 min relative to the start of the albumin infusion (t=0 min) using the equation

$C_{net}\left( t \right)=C_{p}\left( t+15 \right)H\left( t+15 \right)+C_{p}\left( t-25 \right)H\left( t-25 \right)+C_{p}\left( t-180 \right)H\left( t-180 \right)$ (5)

We here assume a “worst case” scenario where the clearances of tracer to and from the extravascular compartment are equal, which, from a mass balance perspective, should represent the maximal TRR possible (meaning that any albumin that is not catabolized will eventually re-enter the circulation). The ratio between plasma and interstitial distribution volumes was assumed to be 1:4, giving a TRR of 3.75% if TER is assumed to be 15%.


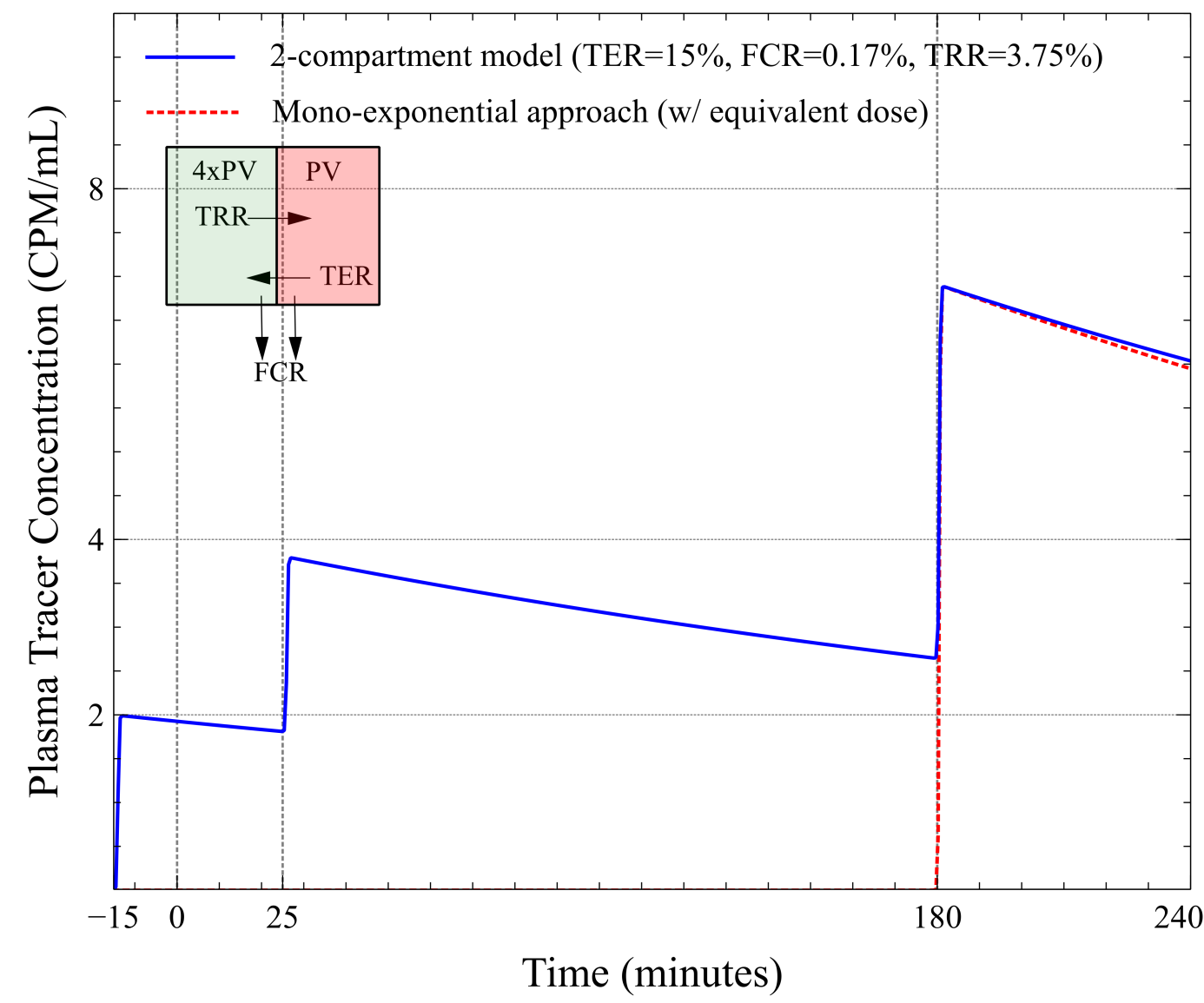


As can be seen in the figure, the mono-exponential approach without re-circulation (red dotted line) gives a small difference (corresponding to a difference in TER <1.0%/h) compared to the model (blue line) even when a large, over-estimated, re-circulation is assumed along with a markedly elevated TER of 15% [[1](#_ENREF_1)]. Simulations were also performed for TER values of 5% and 10% with negligible differences (< 0.1%/h due to a lower TRR). For example, if the difference between a control group (TER 5%/h) and an intervention group is 200% (TER 15%/h) the difference between the two groups may at most be underestimated by 10% due to re-circulation alone. For differences in TER lower than 100% between the groups, re-circulation will have a negligible effect on the difference between the groups.

**References**

1. **Fleck A, Raines G, Hawker F, Trotter J, Wallace PI, Ledingham IM, and Calman KC**. Increased vascular permeability: a major cause of hypoalbuminaemia in disease and injury. *Lancet* 1: 781-784, 1985.

2. **Peters Jr T**. *All about albumin: biochemistry, genetics, and medical applications*. Academic press, 1995.
